# Supplementary material for: Molecular determinants of large cargo transport into the nucleus
Source: eLife. 2020 Jul 21;9:e55963. doi: 10.7554/eLife.55963 (PMC7375812; doi:10.7554/eLife.55963)
Supplement: Supplementary file 1. — We evaluated the appropriateness of a single- vs double-exponential fit to our kinetic data. In this file we report the fit parameters for the double exponential fit with their very high uncertainties and show that their combinations is tightly constrained to values of the mono-exponential fit parameters. [file elife-55963-supp1.docx]

**Supplementary File 1**

|  | **#NLSs** | **A** | **A_stdev_** | **I_MAX1_** | **I_MAX1, stdev_** | **τ_1_** | **τ_1,stdev_** | **I_MAX2_** | **I_MAX2, stdev_** | **τ_2_** | **Τ_2,stdev_** |
| --- | --- | --- | --- | --- | --- | --- | --- | --- | --- | --- | --- |
| **MS2^S37P^** | 0 | 0.19 | 0.01 | 0.13 | 0.01 | 0.13 | 0.03 | 0.68 | 0.26 | 0.006 | 0.00 |
|  | 14 | 0.64 | 0.04 | -0.03 | 15.16 | 0.08 | 8.72 | 0.97 | 15.15 | 0.055 | 0.17 |
|  | 19 | 0.87 | 0.14 | 8.92 | 8.44 | 0.06 | 0.02 | 6.50 | 18.07 | 0.01 | 0.04 |
|  | 23 | 0.52 | 0.22 | 12.71 | 1.50E+04 | 0.06 | 2.31 | 4.24 | 1.50E+04 | 0.04 | 5.80 |
|  | 29 | 0.40 | 0.28 | 27.77 | 3.77E+02 | 0.02 | 0.09 | 6.00 | 3.87E+02 | 0.04 | 0.35 |
|  | 38 | 0.88 | 0.59 | 28.72 | 2.55E+03 | 0.05 | 0.14 | 19.64 | 2.55E+03 | 0.02 | 0.37 |
|  | 54 | 2.04 | 0.39 | 34.09 | 9.21E+03 | 0.03 | 0.26 | 20.79 | 9.23E+03 | 0.03 | 0.62 |
| **I53-47** | 0 | 0.16 | 0.02 | 0.27 | 0.36 | 0.06 | 0.06 | 3.52 | 4.55 | 0.00 | 0.01 |
|  | 15 | 0.53 | 0.12 | 2.75 | 0.20 | 0.14 | 0.02 | 3.71 | 1.09 | 0.01 | 0.01 |
|  | 18 | 3.09 | 0.07 | 1.98 | 0.67 | 0.07 | 0.02 | 9.57 | 25.84 | 0.00 | 0.01 |
|  | 22 | 0.00 | 3.44 | 9.68 | 112.66 | 0.05 | 3.6E-01 | -0.10 | 3.99E+06 | 0.00 | 3.52E+03 |
|  | 22 | 1.88 | 0.23 | 5.29 | 9.68 | 0.04 | 0.05 | 0.60 | 3.18E+03 | 0.00 | 4.28 |
|  | 25 | 2.24 | 0.14 | 2.84 | 2.03 | 0.06 | 0.03 | 0.85 | 7.71E+02 | 0.00 | 0.83 |
|  | 30 | 1.75 | 0.20 | 2.35 | 0.36 | 0.19 | 0.05 | 6.38 | 0.36 | 0.04 | 0.00 |
|  | 35 | 1.23 | 0.29 | 6.70 | 51.21 | 0.03 | 0.11 | 11.25 | 53.48 | 0.05 | 0.08 |
|  | 37 | 0.99 | 0.20 | 3.00 | 2.36E+03 | 0.07 | 3.06 | 3.43 | 2.36E+03 | 0.06 | 2.44 |
|  | 37 | 2.15 | 0.36 | 3.19 | 7.92 | 0.11 | 0.13 | 11.37 | 7.84 | 0.04 | 0.02 |
|  | 41 | 1.07 | 0.23 | 4.90 | 7.68 | 0.01 | 0.04 | 11.97 | 4.51 | 0.06 | 0.02 |
|  | 44 | 0.00 | 0.23 | 9.05 | 5.12E+05 | 0.05 | 34.56 | 1.93 | 5.12E+05 | 0.05 | 162.87 |
| **MS2** | 0 | 0.07 | 0.00 | 0.09 | 0.03 | 0.07 | 0.02 | 1.47 | 7.42 | 0.00 | 0.01 |
|  | 42 | 0.32 | 0.06 | 0.41 | 0.05 | 0.33 | 0.07 | 0.35 | 0.02 | 0.04 | 0.01 |
|  | 54 | 0.10 | 0.07 | 1.04 | 0.07 | 0.30 | 0.04 | 0.15 | 0.08 | 0.06 | 0.03 |
|  | 57 | 0.06 | 0.03 | 0.23 | 1.17E+03 | 0.07 | 13.60 | 1.60 | 1.17E+03 | 0.08 | 2.07 |
|  | 77 | 0.48 | 0.04 | 0.92 | 2.74E+03 | 0.07 | 1.77 | 0.42 | 2.74E+03 | 0.07 | 3.92 |
|  | 86 | 0.52 | 0.04 | 1.95 | 3.43 | 0.05 | 0.04 | 0.86 | 2.75 | 0.02 | 0.08 |
|  | 93 | 0.37 | 0.03 | 1.76 | 0.53 | 0.06 | 0.01 | 1.63 | 13.32 | 0.00 | 0.04 |
|  | 98 | 0.21 | 0.04 | 1.79 | 2.84E+03 | 0.04 | 0.47 | 1.77 | 2.84E+03 | 0.04 | 0.45 |

Notably, although bi-exponential fits provided marginally better fit quality, it was at the expense of very high error bars on the parameter estimates, indicative of over-fitting and “sloppiness” (Gutenkunst, Ryan N., et al. "Universally sloppy parameter sensitivities in systems biology models." *PLoS computational biology* 3.10 (2007)). This is evident in the standard deviation (stdev) recovered from fit for every parameter in the table. Moreover, we observe that despite the large errors on the fitting parameters in the bi-exponential fit, the combinations of these parameters that correspond to parameters in the mono-exponential fit are tightly constrained to values of the mono-exponential fit parameters. Namely, $I_{MAX}\left( mono \right)=I_{MAX1}(bi)+I_{MAX2}(bi)$ and $I_{MAX}\cdot\tau\left( mono \right)=I_{MAX1}\cdot\tau_{1}\left( bi \right)+I_{MAX2}\cdot\tau_{2}(bi)$, as shown in the table below.

|  | **#NLSs** | **I_MAX_ (mono-exponential)** | **I_MAX1_ + I_MAX2_**  **(bi-exponential)** | **I_MAX_* τ (mono-exponential)** | **I_MAX1_*τ1 + I_MAX2_*τ2**  **(bi-exponential)** |
| --- | --- | --- | --- | --- | --- |
| **MS2^S37P^** | 0 | 0.39 | 0.81 | 0.01 | 0.02 |
|  | 14 | 0.94 | 0.94 | 0.05 | 0.05 |
|  | 19 | 12.44 | 15.42 | 0.51 | 0.59 |
|  | 23 | 16.63 | 16.95 | 0.88 | 0.90 |
|  | 23 | 32.88 | 33.77 | 0.74 | 0.77 |
|  | 38 | 43.59 | 48.36 | 1.62 | 1.84 |
|  | 54 | 49.76 | 54.88 | 1.43 | 1.64 |
| **I53-47** | 0 | 2.04 | 3.79 | 0.02 | 0.03 |
|  | 15 | 3.83 | 6.47 | 0.18 | 0.43 |
|  | 18 | 4.22 | 11.55 | 0.17 | 0.17 |
|  | 22 | 3.01 | 9.59 | 0.16 | 0.45 |
|  | 22 | 5.35 | 5.89 | 0.21 | 0.22 |
|  | 25 | 2.86 | 3.70 | 0.19 | 0.17 |
|  | 30 | 7.57 | 8.73 | 0.37 | 0.69 |
|  | 35 | 17.39 | 17.95 | 0.75 | 0.80 |
|  | 37 | 6.45 | 6.43 | 0.41 | 0.41 |
|  | 37 | 14.01 | 14.56 | 0.73 | 0.84 |
|  | 41 | 14.12 | 16.87 | 0.72 | 0.81 |
|  | 44 | 11.01 | 10.98 | 0.51 | 0.52 |
| **MS2** | 0 | 0.25 | 1.56 | 0.00 | 0.01 |
|  | 42 | 0.50 | 0.76 | 0.03 | 0.15 |
|  | 54 | 1.04 | 1.19 | 0.23 | 0.33 |
|  | 57 | 1.83 | 1.83 | 0.14 | 0.14 |
|  | 77 | 1.33 | 1.33 | 0.09 | 0.09 |
|  | 86 | 2.62 | 2.80 | 0.11 | 0.12 |
|  | 93 | 2.09 | 3.39 | 0.10 | 0.11 |
|  | 98 | 3.56 | 3.56 | 0.14 | 0.14 |
